# Prevention of transmission of *Babesia canis* by *Dermacentor reticulatus* ticks to dogs treated orally with fluralaner chewable tablets (Bravecto™)

**DOI:** 10.1186/s13071-015-0923-1

**Published:** 2015-06-04

**Authors:** Janina Taenzler, Julian Liebenberg, Rainer K.A. Roepke, Anja R. Heckeroth

**Affiliations:** MSD Animal Health Innovation GmbH, Zur Propstei, 55270 Schwabenheim, Germany; ClinVet International, Uitsigweg, Bainsvlei, 9338 Bloemfontein, Free State South Africa

**Keywords:** Bravecto™, *Babesia canis*, Babesiosis, Chewable tablets, Fluralaner, *Dermacentor reticulatus*, Dog, Efficacy, Preventive effect, Tick, Tick-borne disease, Transmission blocking

## Abstract

**Background:**

The preventive effect of fluralaner chewable tablets (Bravecto™) against transmission of *Babesia canis* by *Dermacentor reticulatus* ticks was evaluated.

**Methods:**

Sixteen dogs, tested negative for *B. canis* by PCR and IFAT, were allocated to two study groups. On day 0, dogs in one group (*n* = 8) were treated once orally with a fluralaner chewable tablet according to label recommendations and dogs in the control group (*n* = 8) remained untreated. On days 2, 28, 56, 70 and 84, dogs were infested with 50 (±4) *B. canis* infected *D. reticulatus* ticks with tick in situ thumb counts 48 ± 4 h post-infestation. Prior to each infestation, the *D. reticulatus* ticks were confirmed to harbour *B. canis* by PCR analysis. On day 90, ticks were counted and removed from all dogs. Efficacy against ticks was calculated for each assessment time point. After treatment, all dogs were physically examined in conjunction with blood collection for PCR every 7 days, blood samples for IFAT were collected every 14 days and the dog’s rectal body temperature was measured thrice weekly. From dogs displaying symptoms of babesiosis or were PCR positive, a blood smear was taken, and, if positive, dogs were rescue treated and replaced with a replacement dog. The preventive effect was evaluated by comparing infected dogs in the treated group with infected dogs in the untreated control group.

**Results:**

All control dogs became infected with *B. canis*, as confirmed by PCR and IFAT. None of the 8 treated dogs became infected with *B. canis*, as IFAT and PCR were negative throughout the study until day 112. Fluralaner chewable tablet was 100 % effective against ticks on days 4, 30, 58, and 90 and an efficacy of 99.6 % and 99.2 % was achieved on day 72 and day 86 after treatment, respectively. Over the 12-week study duration, a 100 % preventive effect against *B. canis* transmission was demonstrated.

**Conclusions:**

A single oral administration of fluralaner chewable tablets effectively prevented the transmission of *B. canis* by infected *D. reticulatus* ticks over a 12-week period.

## Background

Canine babesiosis, caused by protozoa of the genus *Babesia* through the bite of a vector tick, is a clinically important tick-borne disease. In Europe to date, four *Babesia* species known to affect dogs have been identified. *Babesia canis*, *Babesia vogeli*, *Babesia gibsoni* and *Babesia vulpes* sp. nov.*,* previously known as *Babesia microti*-like [1–3]. Of these species, *B. canis* is the most widely distributed in Europe, coinciding with the distribution of its known vector *Dermacentor reticulatus,* the ornate dog tick. *B. vogeli* is most often found around the Mediterranean basin where *Rhipicephalus sanguineus* is the predominant tick species. The *Babesia vulpes* sp. nov. species seems to be centered in the northwest of Spain whereas the occurrence of *B. gibsoni* is reported more sporadically [2, 4].

*Babesia* spp. are intracellular protozoa habiting in the red blood cells of the host. The clinical signs of babesiosis in dogs vary from mild transient illness to acute disease due to severe haemolysis that rapidly results in death. Clinical findings include pale mucus membranes, elevated body temperature, anorexia, icterus, pyrexia, and splenic and hepatic enlargement [2]. However, the severity of the disease depends on various factors such as the *Babesia* species involved, the age and immune status of the dog and the presence of other infectious diseases [4].

Worldwide, canine babesiosis is one of the most eminent tick-borne diseases [5]. Due to increasing pet ownership, more owners travelling with their pets and the ability of vector arthropods to establish themselves in new localities [6], ticks and tick-borne diseases are spreading throughout the world and are no longer restricted to certain areas.

Once the infected tick has attached to the dog, the risk of pathogen transmission from the tick to the dog is increased by sustained feeding. In most tick-borne disease systems, after initial tick attachment, a feeding period of at least 24 to 48 h is required before transmission of protozoa occur [7]. To prevent the pathogen transmission, it is necessary to kill the infected tick within this time period. To quantify the dynamics of transmission of tick-borne pathogens, transmission blocking tick models have been developed. Such a model includes a sufficient number of treated dogs to test the duration of preventive activity, plus an untreated control group wherein the majority of dogs become infected with the tick-borne pathogen.

In the current study, dogs were treated once orally with fluralaner chewable tablets (Bravecto™). Fluralaner, a new ectoparasiticide in the novel isoxazoline compound class, elicits its primary action through feeding activity, with a duration of efficacy over 12 weeks resulting in the immediate and persistent killing of ticks and fleas on dogs [8]. As fluralaner is a systemic acting ectoparasiticide; its efficacy depends on ticks attaching to the host’s skin and commencing to feed, thereby ingesting the active compound. Due to its rapid speed of killing within 12 h after tick attachment [9], the potential of orally administered fluralaner to prevent *B. canis* transmission was tested in the outlined study.

## Methods

### Study set-up

The study was conducted in accordance with Good Clinical Practice (VICH guideline GL9, Good Clinical Practice, EMA, 2000), and was in compliance with the South African National Standard “SANS 10386:2008: The care and use of animals for scientific purposes” and ethical approval was obtained by the ClinVet Animal Ethics Committee (CAEC) before the study start. The study was performed as a negative controlled, partly blinded, randomized efficacy study.

Sixteen mixed breed dogs (8 males, 8 females) tested negative for babesial DNA by PCR analysis and negative for *B. canis* antibodies (IFAT) before treatment were used. All dogs included were between 1 and 8 years of age and weighed between 13.2 and 26.9 kg. Each dog was in good health; had not been treated with any parasite control product within 3 months prior to a 7-day acclimatization period; did not harbour any ticks before treatment; and was uniquely identified by a microchip number.

Prior to randomization, dogs were clinically examined and weighed. Dogs were ranked within gender by descending body weight and randomly allocated to two study groups (one treatment and one control group) of 8 dogs each using a computer-generated randomization list.

All dogs were kept indoors and housed individually during the study course. Temperature in the dog housing facility ranged between 15.1 and 27.9 °C and the relative humidity between 21.9 and 66.4 %. All dogs were fed a standard commercially available dry dog food once daily and drinking water was provided *ad libitum*.

### Treatment

On day 0 (i.e., day of treatment), dogs in the treatment group were treated once orally with fluralaner chewable tablets (Bravecto™), according to label recommendations. Each dog received half of its daily food ration approximately 20 min before administration of treatment and the balance directly after treatment. The chewable tablet was administered by placement in the back of the oral cavity over the tongue to initiate swallowing. Each treated dog was continuously observed for 1 h after administration to monitor for vomit or tablet spit out, which did not occur in any of the 8 dogs. Dogs in the control group remained untreated. Specific health observations on day 0 were performed hourly, up to 4 h after administration on all dogs (treated and untreated).

### Tick infestations and assessments

A laboratory-bred *D. reticulatus* tick isolate (European origin) infected with *B. canis* was used for each infestation. Tick infestations were conducted on all dogs on days 2, 28 (4 weeks), 56 (8 weeks), 70 (10 weeks), and 84 (12 weeks). One sample of *D. reticulatus* ticks (approximately 50) was taken from each batch of ticks used for each infestation to determine the percentage of infection with *B. canis* by PCR analysis. At each infestation time point, each dog was infested with 50 (± 4) viable, unfed adult *D. reticulatus* ticks (50 % female; 50 % male). Dogs were not sedated for infestation, but during each infestation every dog was placed in an infestation restrainer measuring 90 × 80 × 70 cm (L x W x H) and ticks were manually applied to the animal’s fur; thereafter the animals were restrained for approximately 10 min. During this time ticks which fell off from the animal were re-applied. After 10 min the infestation restrainer was closed, and after 4 h (± 10 min) the dog was released into its cage.

Tick in situ thumb counts were performed on each dog at 48 ± 4 h post each infestation (i.e., on days 4, 30, 58, 72, and 86), but ticks were not removed. On day 90, all remaining ticks on each dog were removed and counted. The personnel conducting tick infestation, tick in situ counting and tick removal on day 90 were blinded to the treatment status of each dog.

### Animal health

To monitor each dog closely for any signs of canine babesiosis, each dog was physically examined by a veterinarian on a 7-day interval until completion of the study (i.e., days 7, 14, 21, 28, 35, 42, 49, 56, 63, 70, 77, 84, 91, 98, 105, and 112). Starting on day 8 after treatment, the rectal body temperature of each dog was measured thrice weekly. General health observations, noting the dog as normal or abnormal, were performed once daily throughout the complete study duration (i.e., starting 7 days prior to treatment until day 112 after treatment). If a dog was noted as abnormal or the rectal body temperature was measured above or equal to 39.4 °C, an additional physical examination by a veterinarian was performed. If one or more parameters were observed as abnormal during this examination, a blood sample for blood smear performance was collected. The blood smear was air dried and stained with a Diff-quick Stain Kit prior to evaluation.

### Blood for serology (IFAT) and PCR analysis

Blood samples for serum analysis for *B. canis* antibodies were collected on a 14-day interval, starting on day 14 after treatment. IFATs were performed using the “MegaScreen® FLUOBABESIA canis” commercial kit. All sera were diluted at 1:80 and were recorded as positive if specific fluorescence was observed, or negative if no fluorescence was observed.

Blood samples for PCR analysis regarding *B. canis* DNA were collected in EDTA tubes from each dog on a 7-day interval. Total genomic DNA was isolated from whole blood samples using a commercial genomic DNA isolation kit (GeneJet Genomic DNA Purification kit, Thermo Scientific). PCR entailed the use of primers Babesia2F (5′-GGAAGGAGAAGTCGTAACAAGGTTTCC-3′) and Bcanis2R (5′-CAGTGGTCACAGACCGGTCG-3′) with combined specificity to the *B. canis* ITS1 region of the DNA in order to amplify a target region of 302 bp [10]. Up to 400 ng isolated DNA served as template for PCR amplification of the target region. PCR products were analysed using agarose gel electrophoresis. A PCR product of approximately 300 bp indicated the presence of the target region in the sample. To verify PCR success in each individual tube, positive, negative, no templates as well as internal amplification controls were included in each run.

### Rescue treatment

A blood sample for blood smear performance was collected from each dog displaying a rectal body temperature above or equal to 39.4 °C, or with a positive PCR analysis result for babesial DNA, or clinical signs of babesiosis observed during physical examination. Dogs confirmed positive for *B. canis* protozoa by blood smear were rescue treated and received the appropriate treatment with diminazene (Berenil; MSD Animal Health) at a dosage of 1 mL/20 kg body weight on the first day and with imidocarb (Forray 65; MSD Animal Health) at a dosage of 1.2 mL/20 kg body weight on the next day. A rescue-treated dog remained part of all health observations (i.e., general health, rectal body temperature assessment, physical examination) but was not subjected to subsequent tick infestations. This dog was moved from the study housing facilities to an outdoor run exposed to ambient environmental conditions, and group housed until final study exclusion. Before moving, all ticks were removed. Blood samples for PCR and IFAT were collected and after confirmation of a babesial infection by both analysis methods, this dog was finally excluded from the study.

### Replacement dogs

Due to the several planned tick infestation time points during the study duration (i.e., tick infestations on days 2, 28, 56, 70, and 84), in addition to the 8 primary included dogs in the control group, replacement dogs were included as required, and to replace animals in this group rescue treated for babesiosis. Before study inclusion, a replacement dog was acclimatized for 7 days prior to first tick infestation for that animal. During this period the dog was tested negative for *B. canis* by PCR analysis and IFAT, and a physical examination by a veterinarian was performed. Replacement dogs were not randomized, but whenever possible the replacement dog had the same sex as the control dog replaced.

### Efficacy evaluation

The statistical analysis was performed using the software package SAS® (SAS Institute Inc., Cary, NC, USA, release 9.3). The individual dog was the experimental unit in all statistical calculations. Data from each tick in situ thumb count time point were analysed separately.

The percentage of efficacy against ticks was calculated for the treatment group at each assessment time point using geometric means with Abbott’s formula:

Efficacy (%) = 100 × (M_C_ - M_T_)/M_C,_ where M_C_ was the mean number of total live attached ticks on untreated control dogs and M_T_ the mean number of total live attached ticks on treated dogs. In case of zero counts, the geometric mean was calculated as follows:

$$ {\mathrm{x}}_{\mathrm{g}}={\left({\displaystyle \prod_{\mathrm{i}=1}^{\mathrm{n}}\left({\mathrm{x}}_{\mathrm{i}}+1\right)}\right)}^{\frac{1}{\mathrm{n}}}-1 $$

Significant differences were assessed between the log-counts of live attached ticks in the treated group at each assessment time point and the log-counts of the untreated control group. Study groups were compared using a linear mixed model including study group as a fixed effect and block as a random effect. The two-sided level of significance was declared when *P* ≤ 0.05.

The percentage of preventive effect against *B. canis* transmission for the treatment group was calculated as follows: Preventive effect (%) = 100 × (T_C_ - T_T_)/T_C_, where T_C_ is the total number of infected dogs in the untreated group and T_T_ is the total number of infected dogs in the treated group. A dog was regarded infected with *B. canis*, if it was tested serologically positive for *B. canis* antibodies (IFAT) and positive for *B. canis* DNA in PCR assay.

## Results

No treatment-related adverse events were observed in any of the 8 dogs treated orally with fluralaner during the 12-week post-treatment observation period. In total 19 replacement dogs (10 male, 9 female) were included in the control group throughout the study, ensuring that at each tick infestation time point, the control group consisted of 8 animals, which was possible for all infestation time points except the last one on day 84. For tick challenge on day 84 only 6 control animals were available, from which two were tested positive by blood smear and PCR analysis on day 85 and rescue treated, so that for tick in situ thumb counting on day 86 the control group consisted of 4 animals. Efficacy calculation for day 86 and day 90 were therefore calculated with 4 control dogs. The mean tick counts and the detailed efficacy against ticks are shown in Table 1. An efficacy against ticks at each assessment time point between 99.2 and 100 % was achieved after single oral fluralaner treatment.

**Table 1:** Mean tick counts and efficacy against ticks after single oral treatment with fluralaner chewable tablets

Day 4	Mean tick counts^a^ (control/treated) [n]	18.6/0.0
	Efficacy [%]	100^b^
Day 30	Mean tick counts^a^ (control/treated) [n]	18.8/0.0
	Efficacy [%]	100^b^
Day 58	Mean tick counts^a^ (control/treated) [n]	12.7/0.0
	Efficacy [%]	100^b^
Day 72	Mean tick counts^a^ (control/treated) [n]	22.2/0.1
	Efficacy [%]	99.6^b^
Day 86	Mean tick counts^a^ (control/treated) [n]	23.0/0.2
	Efficacy [%]	99.2^b^
Day 90	Mean tick counts^a^ (control/treated) [n]	22.3/0.0
	Efficacy [%]	100^b^

^a^Geometric mean live attached ticks

^b^Log-counts of ticks from the treated group were significantly different (*p* < 0.0001) from log-counts of the untreated control group

None of the dogs treated with Bravecto™ chewable tablets developed any clinical signs referring to babesiosis. Dogs in the control group developed clinical signs referring to babesiosis as pale mucous membranes, rectal body temperature above or equal to 39.4 °C, depressed/listless general behaviour, enlarged lymph nodes and enlarged spleen.

Rectal body temperatures were measured for each dog thrice weekly. In 19 of 27 control dogs a rectal body temperature above or equal to 39.4 °C was measured on at least one measurement time point during the study. In the treated group in 1 of 8 dogs a rectal body temperature above or equal to 39.4 °C was measured once 17 days after treatment. This elevated rectal body temperature was not confirmed to be related to an infection with *B. canis,* as blood analysis results (blood smear, PCR and IFAT) for this animal were negative for *B. canis* throughout the study period (see Table 2).

**Table 2:** Number of dogs with increased rectal body temperature (RBT) and number of dogs tested positive for *B. canis* via blood smear, PCR and IFAT

Study group	RBT	Blood smear	PCR	IFAT
	≥39.4 °C	[POS]	[POS]	[POS]
Untreated control [n]	19/27	27/27	27/27	27/27
Treatment group^a^ [n]	1^b^/8	0/8	0/8	0/8

^a^oral treatment with fluralaner chewable tablets

^b^one dog had a RBT of 39.8 °C once 17 days after treatment

At each infestation time point, 12–16 % of ticks were found to be infected with *B. canis* by PCR analysis. The infection model was regarded as valid as all dogs in the untreated control group were infected with *B. canis*, as confirmed positive for *B. canis* by blood smear; for babesial DNA by PCR analysis; and for *B. canis* antibodies by IFAT after first or subsequent tick infestation. None of the treated dogs became infected with *B. canis* during the complete study duration, as confirmed by the absence of *B. canis* antibodies in the IFAT and a negative test result for babesial DNA by PCR analysis on any of the scheduled blood analysis time points up to 4 weeks after the last tick infestation (see Table 2). A 100 % preventive effect against the transmission of *B. canis* by infected *D. reticulatus* ticks was achieved after single oral fluralaner treatment (see Table 3).

**Table 3:** Preventive effect against the transmission of *B. canis* by *D. reticulatus* ticks

	Untreated control	Treatment group^a^
Infected [n]	27/27	0/8
Non-infected [n]	0/27	8/8
Infected [%]	100	0
Prevention [%]	-	100
*p*-Value	-	0.0002

^a^oral treatment with fluralaner chewable tablets

## Discussion

The blocking of pathogen transmission (preventive effect) to dogs through the bite of vector ticks has become an increased demand of pet owners and veterinarians in the evaluation of the capacity of anti-tick compounds.

Canine babesiosis is one of the clinically most significant and eminent tick-borne diseases [5], and was therefore used as study model to determine the ability of fluralaner to prevent the transmission of *B. canis* by infected *D. reticulatus* ticks. *B. canis* protozoa infect the red blood cells, causing mild to severe disease with different severities in clinical signs until death, if untreated. For this reason, dogs in the untreated control group were immediately rescue treated after they had been tested positive by blood smear. For these rescue-treated dogs, replacement dogs were included, to maintain a sufficient number of at least 6 dogs in the control group for statistical analysis, as required by the guideline for evaluating the efficacy of parasiticides for the treatment, prevention and control of flea and tick infestations on dogs and cats [11].

Fluralaner is the first orally-administered compound leading to systemic activity with an efficacy duration over 12 weeks against ticks and fleas [8, 12]. Until 2014, tick control compounds for dogs were available as spot-ons, sprays or collars exhibiting its tick-killing efficacy via blood meal and/or contact exposure/repellency [13]. In the speed of kill studies from Wengenmayer *et al.* [9], it was demonstrated that orally-administered fluralaner starts to kill ticks present on the dog as early as 4 h (89.6 %), showing almost complete tick-killing efficacy within 12 h after treatment over the entire 12-week period of efficacy. These results are confirmed in this study by the excellent efficacy results against ticks (see Table 1). As fluralaner elicits its primary action through feeding activity, the protective effect of fluralaner is less obvious. The efficacy of fluralaner depends on ticks attaching to the host’s skin and commencing feeding, thereby ingesting the active compound before being killed [8]. The transmission time of *B. canis* from infected *D. reticulatus* ticks is given by Heile *et al.* [14] with 48–72 h after tick attachment. The tick’s attachment to the host’s skin starts the maturation of the sporozoites located in the salivary glands of the tick. A few days later after the tick has attached, pathogen transmission through the saliva from the tick causes host infection [15]. Due to its rapid tick-killing effect, fluralaner effectively prevented the transmission of *B. canis* from infected *D. reticulatus* ticks to the dogs (Table 3). Fluralaner chewable tablets demonstrated an efficacy against ticks between 99.2 and 100 % over the entire 12-weeks study duration.

An active ingredient with a longer re-treatment interval such as fluralaner reduces the risk of treatment failure as a consequence of poor owner compliance with monthly treatment recommendations. Owner compliance is an important component of successful control and prevention of tick infestations during tick season. This study demonstrated that treatment with fluralaner chewable tablet is not only effective against ticks and protects the dog against pathogen transmission, but also remains effective over a 12-week period following treatment. Moreover, in addition to its efficacy against *D. reticulatus,* fluralaner is effective for the same period of time against other ticks and fleas that may concomitantly infest these animals [12, 16].

## Conclusions

Single oral administration of fluralaner chewable tablets (Bravecto™) to dogs prevented the transmission of *B. canis* by infected *D. reticulatus* ticks by 100 % over 12 weeks. An efficacy against ticks between 99.2 and 100 % was achieved over the entire 12-week study duration. The long re-treatment interval of fluralaner chewable tablets offers more convenience over monthly tick-control treatments, with a potential compliance advantage.
